# Is preoperative glenoid bone mineral density associated with aseptic glenoid implant loosening in anatomic total shoulder arthroplasty?

**DOI:** 10.1186/s12891-020-03892-0

**Published:** 2021-01-08

**Authors:** Sandrine Mariaux, Raphaël Obrist, Alain Farron, Fabio Becce, Alexandre Terrier

**Affiliations:** 1 Service of Orthopedics and Traumatology, Lausanne University Hospital and University of Lausanne, Avenue Pierre-Decker 4, 1011 Lausanne, Switzerland; 2 Laboratory of Biomechanical Orthopedics, Ecole Polytechnique Fédérale de Lausanne, Station 9, 1015 Lausanne, Switzerland; 3 Department of Diagnostic and Interventional Radiology, Lausanne University Hospital and University of Lausanne, Rue du Bugnon 46, 1011 Lausanne, Switzerland

**Keywords:** Anatomic total shoulder arthroplasty, Aseptic loosening, Bone mineral density, Computed tomography, Glenoid implant

## Abstract

**Background:**

Aseptic loosening of glenoid implants is the primary revision cause in anatomic total shoulder arthroplasty (aTSA). While supported by biomechanical studies, the impact of glenoid bone quality, more specifically bone mineral density (BMD), on aseptic glenoid loosening remains unclear. We hypothesized that lower preoperative glenoid BMD was associated with aseptic glenoid implant loosening in aTSA.

**Methods:**

We retrospectively included 93 patients (69 females and 24 males; mean age, 69.2 years) who underwent preoperative non-arthrographic shoulder computed tomography (CT) scans and aTSA between 2002 and 2014. Preoperative glenoid BMD (CT numbers in Hounsfield unit) was measured in 3D using a reliable semi-automated quantitative method, in the following six contiguous volumes of interest (VOI): cortical, subchondral cortical plate (SC), subchondral trabecular, and three successive adjacent layers of trabecular bone. Univariate Cox regression was used to estimate the impact of preoperative glenoid BMD on aseptic glenoid implant loosening. We further compared 26 aseptic glenoid loosening patients with 56 matched control patients.

**Results:**

Glenoid implant survival rates were 89% (95% confidence interval CI, 81–96%) and 57% (41–74%) at 5 and 10 years, respectively. Hazard ratios for the different glenoid VOIs ranged between 0.998 and 1.004 (95% CI [0.996, 1.007], *p*≥0.121). Only the SC VOI showed significantly lower CTn in the loosening group (622±104 HU) compared with the control group (658±88 HU) (*p*=0.048), though with a medium effect size (d=0.42). There were no significant differences in preoperative glenoid BMD in any other VOI between patients from the loosening and control groups.

**Conclusions:**

Although the preoperative glenoid BMD was statistically significantly lower in the SC region of patients with aseptic glenoid implant loosening compared with controls, this single-VOI difference was only moderate. We are thus unable to prove that lower preoperative glenoid BMD is clearly associated with aseptic glenoid implant loosening in aTSA. However, due to its proven biomechanical role in glenoid implant survival, we recommend extending this study to larger CT datasets to further assess and better understand the impact of preoperative glenoid BMD on glenoid implant loosening/survival and aTSA outcome.

**Supplementary Information:**

The online version contains supplementary material available at 10.1186/s12891-020-03892-0.

## Background

The incidence of total shoulder arthroplasty (TSA) is increasing with population growth, aging, and the concomitant increase in prevalence of glenohumeral osteoarthritis (OA) [1–4]. Since the 1970s and the implantation of the first contemporary total shoulder prostheses, issues with radiolucent lines around glenoid components and aseptic loosening have been observed [5–7]. Despite major technological advances in prosthesis design, aseptic loosening of glenoid implants remains the primary cause of revision surgeries in anatomic TSA (aTSA) – affecting 0.01 to 6% of shoulder replacement surgeries – and its trend is increasing in proportion to the increase in TSA [5, 7–9].

The main risk factors for aseptic loosening of glenoid implants reported in the literature are related to biomechanical causes: malpositioning (in particular retroversion) of the glenoid component, head/glenoid implant diameter mismatch, persistence/recurrence of static posterior subluxation of the humeral head, and rotator cuff deficiency [10–14]. Indeed, all the above mentioned factors can cause impingements and/or excessive stress on the glenoid implant or at its bone interface. This may eventually lead to repeated micromotion and progressive implant loosening.

In addition to the aforementioned potential biomechanical causes of aseptic glenoid loosening in aTSA, glenoid bone quality has also been suggested and investigated as an additional contributor. Lower bone mineral density (BMD) has indeed been shown to increase the respective risks of periprosthetic fracture in total ankle arthroplasty [15] and pedicle screw loosening in spinal fusion surgery [16]. However, the precise role of preoperative glenoid BMD in aseptic glenoid loosening has not been thoroughly evaluated using a reliable quantitative CT method so far [17–22].

Terrier et al. assessed the association between CT-derived preoperative glenoid BMD and several biomechanical predictions (cement stress, bone-cement interface stress, and bone strain) in aTSA, using patient-specific finite element models [22]. They showed that preoperative glenoid BMD was strongly negatively correlated with these biomechanical predictions, particularly in the subchondral trabecular bone, thus suggesting that this factor may contribute to aseptic loosening of glenoid implants [22]. However, to our knowledge, the clinical impact and relevance of the patient’s preoperative glenoid BMD on glenoid implant survival in aTSA has not been evaluated in large case series yet.

Therefore, our objective was to test the hypothesis that aseptic loosening of glenoid implants in aTSA is associated with lower preoperative glenoid BMD. To do so, we retrospectively analyzed an institutional case series of aTSA patients, quantified the preoperative glenoid BMD in several predefined volumes of interest (VOI) from shoulder CT datasets, and evaluated its association with aseptic glenoid loosening.

## Methods

### Patients and study design

After approval by the institutional ethics committee (Lausanne University Hospital CER-VD, protocol 136/15), we retrospectively reviewed all consecutive cases treated with aTSA in our tertiary referral hospital between January 2002 and December 2014 (*n*=262). All patients were operated through a deltopectoral approach by the same senior shoulder surgeon for the following clinical indications: primary glenohumeral OA (*n*=200), post-traumatic glenohumeral OA (*n*=31), avascular necrosis of the humeral head (*n*=11), inflammatory arthritis (*n*=12), or another diagnosis (*n*=8). The cemented Aequalis all-polyethylene keeled glenoid component (Wright-Tornier, Bloomington, MN, USA) was implanted after minimum glenoid bone reaming to preserve the bone stock. Holes for keeled glenoid implants were drilled using proper instrumentation, and high-viscosity bone cement was vacuum mixed and applied to fix them. All glenoid implants (small, medium, or large) were adapted to the size of the patient’s glenoid cavity according to the manufacturer’s recommendation chart for heads/glenoids diameter mismatch.

Of these 262 cases, we included all patients who had undergone preoperative shoulder CT scans (*n*=184). The following exclusion criteria were subsequently applied: patients lost to follow-up (< 2 years) (*n*=51), shoulder CT arthrography (*n*=32), non-arthrographic shoulder CT scans with metal artifacts or incomplete CT coverage of the glenoid (*n*=3), septic loosening of the glenoid implant (*n*=3), glenoid implant malpositioning (*n*=1), and recurrent postoperative scapulohumeral subluxation (*n*=1). CT arthrograms were excluded because the intra-articular iodinated contrast medium interfered with glenoid BMD measurements due to beam hardening artifacts [23, 24]. The resulting study population consisted of 93 patients, with a mean age at surgery of 69.2 years (range, 45.9–88.2 years), a female/male ratio of 69/24, a mean body mass index (BMI) of 27.4 (range, 18.4–42.4), and a smoking history in 10/93 patients (10.8%).

### Shoulder CT protocol

All preoperative non-arthrographic shoulder CT scans were performed on 8-, 16-, or 64-detector row CT systems (LightSpeed Ultra, LightSpeed Pro 16, LightSpeed VCT, and Discovery CT750 HD; GE Healthcare, Waukesha, WI, USA) using the following standardized data acquisition settings: tube potential, 120–140 kVp; tube current, 144–440 mA; and gantry revolution time, 0.5–0.8 s. The image reconstruction parameters were as follows: field of view 14× 14–32× 32 cm (thus yielding in-plane pixel sizes of 0.27× 0.27–0.63× 0.63 mm); section thickness, 0.6–3.0 mm; section interval, 0.3–2.0 mm; and sharp (bone or bone plus, GE Healthcare) kernels.

### CT assessment of preoperative glenoid BMD and morphology

**Fig. 1:**
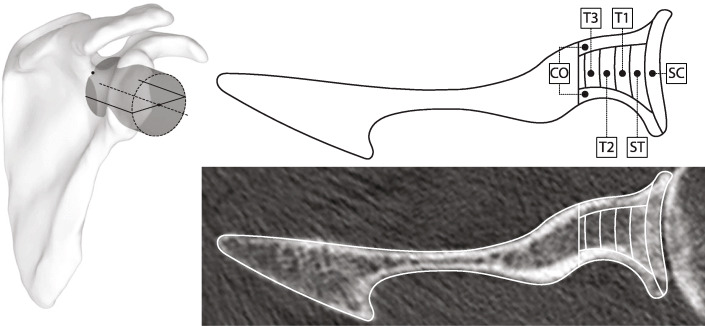
Computed tomography numbers (in Hounsfield unit) were measured in six contiguous 3-mm-thick volumes of interest: cortical bone (CO), subchondral cortical plate (SC), subchondral trabecular bone (ST), and three successive adjacent layers of trabecular bone (T1, T2, and T3). All volumes of interest were defined within a 40-mm-high cylinder aligned with the mediolateral scapular axis, centered on and adjusted to include the entire glenoid cavity, and whose medial base was positioned at the spinoglenoid notch

Preoperative glenoid BMD was quantified in 3D from preoperative shoulder CT datasets using the same reliable method described in detail elsewhere [22]. Briefly, we measured the average CT numbers (CTn; in Hounsfield unit, HU) in six contiguous 3-mm-thick VOIs (cortical bone (CO), subchondral cortical plate (SC), subchondral trabecular bone (ST), and three successive adjacent layers of trabecular bone (T1, T2, and T3)) defined within a 40-mm-high cylinder aligned with the mediolateral scapular axis, centered on and adjusted to include the entire glenoid cavity, and whose medial base was positioned at the spinoglenoid notch (Fig. 1). Within this cylinder fully encompassing the glenoid, bone mineral tissue was then segmented using a lower threshold of 300 HU [25]. All these measurements were performed using the Amira software (Thermo Fisher Scientific, Waltham, MA, USA) and user-defined Matlab scripts (MathWorks, Natick, MA, USA).

In addition, preoperative glenoid morphology was assessed on a picture archiving and communication system workstation (Vue PACS; Carestream Health, Rochester, NY, USA) by a board-certified orthopedic surgeon and a senior musculoskeletal radiologist independently, using the updated Walch grading system [26]. In the case of discrepancy, consensus was reached with a senior shoulder surgeon.

### Aseptic loosening of glenoid implants

Aseptic loosening of glenoid implants was assessed using conventional shoulder radiographs (anteroposterior and axial/axillary, with/without lateral/scapular “Y” views) performed at regular follow-up visits at 3, 6, 12, and 24 (+/− 1) months, followed by once a year or every 2 years, depending on the patient’s clinical course. In the case of radiological uncertainty and/or onset of clinical symptoms (e.g. pain, feeling of instability or locking, decreased range of motion), shoulder CT arthrography was performed. Radiographic prosthetic loosening was defined as the presence and/or enlargement over time of complete (thickness > 1.5 mm) radiolucent lines at the glenoid bone-cement interface, and/or migration (> 5 mm), tilt (> 5°), or shift of the glenoid component [2, 5, 8, 27, 28]. The same observers as above independently reviewed all shoulder radiographs and, when available (*n*=11), postoperative CT arthrograms, with the same consensus agreement approach in the case of discrepancy.

### Statistical analysis

We first performed a Kaplan-Meier estimator to evaluate the survival (radiographic aseptic loosening as described above) of glenoid implants in the overall aTSA study population. Survival rate was assessed at 5 and 10 years postoperatively. This analysis was supplemented with univariate Cox proportional hazards regression models to estimate the effect of preoperative glenoid BMD (reflected by CTn in the various predefined VOIs) on glenoid implant survival (defined by the absence of aseptic glenoid loosening). We further analyzed the influence of gender, age, BMI, and smoking history on implant survival.

In a second step, we compared two patient groups. All patients with glenoid implant aseptic loosening were included in the loosening (LSG) group, while the control (CTR) group was formed by matching patients in terms of follow-up time, gender, age, BMI, smoking history, and Walch glenoid types. The follow-up time in the CTR group was at least 2 years. Continuous and categorical variables were compared using one-tailed independent-samples Student’s t-test (or Wilcoxon signed-rank test in the case of skewed distribution with Lilliefors test) and chi-squared test, respectively. We tested the hypothesis that preoperative glenoid BMD (CTn) was numerically lower in the LSG than in the CTR group. Statistical significance was set at *p*< 0.05. The effect size was measured using Cohen’s d standardized difference between the two means, and interpreted as small (d≤0.2), medium (d=0.5), or large (d≥0.8) [29]. Statistical analysis was performed using Matlab’s Statistics and Machine Learning Toolbox (MathWorks) by one of the authors (AT).

## Results

**Fig. 2:**
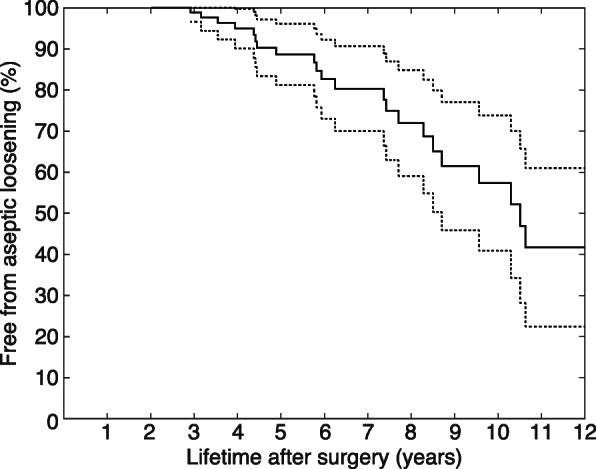
Kaplan-Meier survival curve (solid line) for glenoid implants in patients with anatomic total shoulder arthroplasty, with the corresponding 95% confidence intervals (dashed lines)

The average follow-up time for the 93 patients included in our study was 6.2 years (range, 2.0–14.1 years). Among them, 26 (28.0%) developed aseptic loosening of their glenoid implant according to our diagnostic criteria described above, which was confirmed in all cases intraoperatively. The Kaplan-Meier survival curve showed glenoid implant survival rates (absence of radiological loosening) of 89% (95% confidence interval (CI), [81, 96%]) and 57% (95% CI, [41, 74%]) at 5 and 10 years, respectively (Fig. 2). The hazard ratios (HR) of Cox regressions for the different VOIs were very close to 1 (95% CI, [0.996, 1.007]). Gender (HR, 0.900 (95% CI, [0.368, 2.201]), *p*=0.817), age (HR, 1.001 (95% CI, [0.952, 1.053]), *p*=0.966), BMI (HR, 1.044 (95% CI, [0.957, 1.139]), *p*=0.332), and smoking history (HR, 0.364, (95% CI, [0.049, 2.717]), *p*=0.325) were also not significantly associated with aseptic glenoid loosening (Supplemental material).

The LSG group consisted of 26 patients: 19 females, 7 males; mean age, 67.5 years (range, 49.7–80.9 years); mean BMI, 26.8 kg/m^2^ (range, 18.4–40.9 kg/m^2^) (Table 1). Walch glenoid types were as follows: A1 (*n*=4), A2 (*n*=8), B1 (*n*=4), and B2-B3 (*n*=10). The mean follow-up time was 7.6 years (range, 2.9–14.1 years). On the other hand, the following 59 patients matched for follow-up time, gender, age, BMI, smoking history, and Walch glenoid types were included in the CTR group: 43 females, 16 males; mean age, 69.5 years (range, 45.9–88.2 years); mean BMI, 27.3 kg/m^2^ (range, 19.1–41.2 kg/m^2^) (Table 1). Walch glenoid types were as follows: A1 (*n*=7), A2 (*n*=15), B1 (*n*=17), B2-B3 (*n*=16), C (*n*=3), and D (*n*=1). The mean follow-up time in this CTR group was 6.1 years (range, 3.1–13.2 years).

**Table 1:** Comparison of patient demographics and characteristics between the loosening (LSG) and control (CTR) groups

	LSG (***n***=26)	CTR (***n***=59)	***P***-value
**Gender**	19 F / 7 M	43 F / 16 M	0.985
**Age (years)**	67.5 (49.7–80.9)	69.5 (45.9–88.2)	0.362
**Body mass index (kg/m**^**2**^**)**	26.8 (18.4–40.9)	27.3 (19.1–412)	0.667
**Walch glenoid type**	A1 (*n*=4), A2 (*n*=8), B1 (*n*=4), B2-B3 (*n*=10)	A1 (*n*=7), A2 (*n*=15), B1 (*n*=17), B2–3 (*n*=16), C (*n*=3), D (*n*=1)	0.644
**Follow-up time (years)**	7.6 (2.9–14.1)	6.1 (3.1–13-2)	0.055

**Fig. 3:**
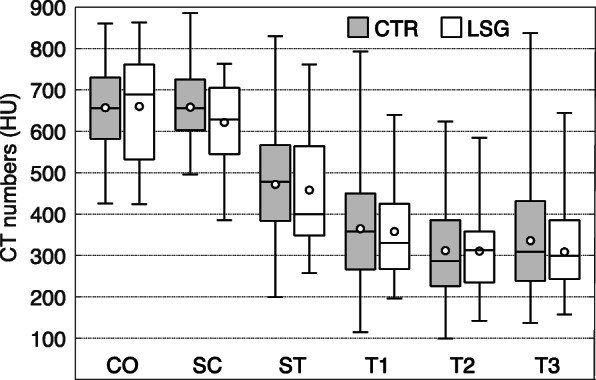
Box-and-whisker plot showing CT numbers (in Hounsfield unit, HU) for each of the six volumes of interest (cortical bone (CO), subchondral cortical plate (SC), subchondral trabecular bone (ST), and three successive adjacent layers of trabecular bone (T1, T2, and T3)) in patients with aseptic loosening of glenoid implants (LSG) compared with control (CTR) patients. Circles represent mean values

CTn were normally distributed in all different glenoid VOIs, except in ST of the LSG group. Only the SC VOI showed significantly lower CTn in the LSG group (622±104 HU) compared with the CTR group (658±88 HU) (*p*=0.048), though with a medium effect size (d=0.420). There were no significant differences in CTn in any other VOI between patients from the LSG and CTR groups (Fig. 3 and Table 2).

**Table 2:** Average CT numbers (in Hounsfield unit) for each of the six volumes of interest (cortical bone (CO), subchondral cortical plate (SC), subchondral trabecular bone (ST), and three successive adjacent layers of trabecular bone (T1, T2, and T3)) in patients from the loosening (LSG) and control (CTR) groups, with the corresponding standard deviations (SD), minimum (Min) and maximum (Max) values, Cohen’s d effect sizes, and *p*-values

	CO		SC		ST		T1		T2		T3	
	LSG	CTR	LSG	CTR	LSG	CTR	LSG	CTR	LSG	CTR	LSG	CTR
**Average**	660	657	622	658	458	472	358	365	310	312	309	338
**SD**	124	104	104	88	138	135	115	139	105	118	105	137
**Min**	424	426	385	496	257	199	196	115	142	99	157	137
**Max**	853	861c	763	886	762	830	640	793	584	624	645	837
**Cohen’s d**	0.029		0.420		0.102		0.049		0.001		0.197	
***P*-value**	0.547		0.048		0.224		0.413		0.483		0.187	

## Discussion

We hypothesized that lower preoperative glenoid BMD was associated with aseptic loosening of glenoid implants in aTSA. Although the preoperative glenoid BMD was statistically significantly lower in the SC region of patients with aseptic glenoid implant loosening compared with controls, this single-VOI difference between groups had only a moderate effect size. We were thus unable to prove that lower preoperative glenoid BMD is significantly associated with aseptic glenoid implant loosening in aTSA.

CT has previously been used to characterize the quality of the glenoid bone support and its relationship with aseptic glenoid implant loosening in aTSA. Chevalier et al. used micro finite element models based on micro-CT scans of cadaveric scapulae to evaluate the influence of bone volume fraction, trabecular anisotropy and cortical thickness on stress within the periprosthetic bone and cement mantle [19]. In a further computational study, Chen et al. measured the glenoid BMD in HU after simulated eccentric reaming for version correction of Walch B2 glenoids [18]. They analyzed BMD in five adjacent 1-mm layers under the reamed glenoid surface, and concluded that increased version correction resulted in gradual depletion of high-quality bone from the anterior regions of B2 glenoids. Chamseddine et al. recently measured the glenoid BMD using a clinical quantitative CT technique on cadaveric scapulae where keeled or pegged cemented glenoid components were implanted [17]. They divided the glenoid in different regions (inferior or superior, and inner, peripheral or full regions) and quantified BMD in mg of calcium hydroxyapatite per cm^3^. They reported that glenoids with lower BMD exhibited increased micromotion and displacement at the bone-implant interface, suggesting that implant failure most likely occur in glenoids with lower BMD, and that the fixation design may play a secondary role.

Other author groups have investigated and compared the glenoid BMD in various shoulder disorders. Couteau et al. initially compared the glenoid BMD in HU in patients with rotator cuff disorders, primary glenohumeral OA, and rheumatoid arthritis using CT datasets and subdividing glenoids into 20 VOIs [30]. They found that the glenoid BMD was higher centrally in patients with rotator cuff disorders, as opposed to primary OA where BMD was higher posteriorly and inferiorly. Divergent results were reported more recently by Harada et al. who reported, using CT osteoabsorptiometry in seven glenoid areas, a decrease in subchondral BMD in the central glenoid region of shoulders with symptomatic rotator cuff tears [31]. This variation in glenoid BMD was further emphasized by Knowles et al. who compared CT-based regional BMD and porosity in symmetric and asymmetric OA glenoid erosion patterns [32]. They subdivided glenoids in quadrants and two different 2.5-mm depths. Concentric OA glenoids exhibited uniform subarticular BMD, while eccentric OA glenoids (Walch B2-B3 types) showed densest bone with least porosity postero-inferiorly or in the neoglenoid region. Simon et al. reported similar results, while quantifying and characterizing in 3D the glenoid subchondral BMD with CT in five zones and three layers/depths [33]. They found that glenoid BMD varied depending on depth from the articular surface, topographic zone, and OA wear pattern. All these studies confirm the importance of evaluating glenoid BMD in several specific regions/volumes rather than considering only one average density/CTn, when assessing its impact on aseptic loosening. However, none of these studies has yet investigated the association between preoperative glenoid BMD and aseptic loosening of glenoid implants in aTSA.

Our results confirm that the preoperative glenoid BMD gradually decreases with distance/depth from the articular surface. As expected and previously reported, cortical bone regions were denser than trabecular bone regions [18, 32, 33]. We also observed that the SC BMD was lower in patients with aseptic glenoid implant loosening compared with CTR cases. It is unfortunately difficult to compare our results in terms of CTn (HU)/BMD with other studies because the measured glenoid regions/VOIs varied greatly among studies. Moreover, the vast majority of previous studies were biomechanically oriented, using computerized or cadaveric models, as opposed to ours which focused primarily on clinical outcome, more specifically aseptic loosening of glenoid implants. In our study, we first divided the glenoid into cortical and trabecular bone. Indeed, we avoided mixing cortical and trabecular bone, assuming that they could reasonably have different biomechanical properties and effects on the glenoid bone support. For the same reason, we subdivided cortical bone into eccentric and articular regions/VOIs, and trabecular bone into several contiguous layers at different depths from the articular surface. We then used CTn in HU as a surrogate for BMD, as previously proven in the literature. Jun et al. recently analyzed with clinical CT imaging the architecture and mineralization of cadaveric glenoids compared to high-resolution micro-CT. They showed that clinical CT imaging was able to quantify regional (anatomic and peri-implant) variations in glenoid BMD [34]. Previously, Schreiber et al. demonstrated that CTn in HU correlated well with BMD and compressive strengths as measured with dual x-ray absorptiometry scans and mechanical testing of synthetic bone models [35]. Most recently, a CT study reported that OA glenoids had a higher BMD than normal glenoids, with higher CTn in the SC region particularly posteriorly, which is consistent with our findings [36].

Our study shows a 28% rate of aseptic glenoid implant loosening at 6.2 years and 57% survival rate at 10 years follow-up, which differ from the rates reported in the literature. In a review, Gonzalez et al. found a 14.3% rate of aseptic loosening in a series of 2657 aTSAs with a mean follow-up of 6 years [37]. However, Walch et al., in a multicenter study based on radiographic analysis [38], and Denard et al. both reported higher aseptic loosening rates of 32 and 43% at mean follow-ups of 8.5 and 9.5 years, respectively; the latter study in patients aged 55 years or younger [39]. On the other hand, the estimated survival rate of aTSA is 83–95% at 10 years [5, 40, 41]. However, the glenoid implant survival rate reported in our study is biased for several reasons. First, our study included B2-B3 glenoids and patients with inflammatory arthritis, which could increase complication rates. Indeed, the indications for TSA have changed and narrowed over time [42]. Second, we had to exclude a substantial number of patients because they did not underwent preoperative shoulder CT scans or only shoulder CT arthrograms which could not be used here. All this had an influence on our reported lower 10-year glenoid implant survival rate.

To our knowledge, our study is the first to quantitatively assess the impact of preoperative glenoid BMD in 3D on glenoid implant survival – more specifically aseptic glenoid loosening – in aTSA. Most previous studies have evaluated the quality of the glenoid bone support using CT datasets (either micro- or conventional CT), but only few have correlated their findings with clinical and radiological outcomes [17–19, 22]. Our semi-automated quantitative measurement method based on a computerized 3D scapular reconstruction model has proven its reliability and already helped improving glenoid implant positioning [43]. This method further allows an in-depth analysis with subdivision of the glenoid bone and its region-specific BMD, distinguishing between the various cortical and trabecular regions. The technique is being made fully automated using deep learning, which should enable future rapid analysis of large clinical CT datasets.

Among the major limitations of our study are the inherent weaknesses of a retrospectively designed study and the relatively high number of patients lost to follow-up. Furthermore, the minimum follow-up was only 2 years, which may have underestimated cases with aseptic glenoid implant loosening occurring at mid- or long-term. However, the average follow-up time for LSG and CTR groups was longer and appropriate. Although aseptic glenoid implant loosening is one of the main complications in aTSA, the prosthetic survival rate is estimated to range between 95 and 99% and 83–95% at 5 and 10 years, respectively [5, 41, 44]. Despite recommendations for regular follow-up, patients with favorable clinical outcome tend not to show up at scheduled follow-up visits. Another study limitation is the lack of clear definition and consensus for the diagnosis of aseptic glenoid implant loosening. The most widely used definition from Cofield states that aseptic loosening is a shift and medialization of the glenoid component usually accompanied by superior tilting of the glenoid prosthetic surface on plain radiographs [45, 46]. A more recent definition by Martin et al. defined aseptic loosening as migration of more than 5 mm or tilt of more than 5 degrees of the glenoid implant [28]. However, smaller displacements or enlargements over time of radiolucent lines at the bone-cement interface may also be seen in patients with aseptic glenoid implant loosening [28]. Furthermore, the distinction between radiolucent lines around the glenoid implant, which are relatively common (10–94%) and usually asymptomatic [27, 47, 48], and aseptic loosening remains unclear. A further limitation is the absence of systematic postoperative shoulder CT scans to assess both the accurate position of the glenoid implant relative to the glenoid VOIs measured preoperatively, and the glenoid bone-cement interface postoperatively. Indeed, depending on intraoperative glenoid bone reaming (patients with B2-B3 glenoid types all underwent minimum anterior reaming in our series, *n*=7), the bone region on which the glenoid implant was lying may be slightly different/offset from the corresponding VOI measured on preoperative CT. Our results should also be weighed by missing retrospective data on and analysis of potential confounding factors for aseptic glenoid implant loosening, such as patients’ comorbidities (e.g. diabetes) and activity level. Finally, the type of glenoid implant used may also have an influence on our results. During the study inclusion period, only keeled glenoid implants were implanted in our institution. Recent data show that such glenoid implant designs are associated with a slightly increased rate of aseptic loosening compared with pegged implants [49].

## Conclusions

Although we observed lower preoperative glenoid BMD in the SC region of patients with aseptic glenoid implant loosening compared with matched CTR cases, this statistical difference was only moderate (medium effect size) and not found in any other glenoid regions. We were thus unable to assert that lower preoperative glenoid BMD is clearly associated with aseptic loosening of glenoid implants in aTSA. However, due to the proven biomechanical role of the bone support in glenoid implant survival, we still recommend extending this initial study to larger CT datasets with higher follow-up rates to further assess and better define the real impact of the preoperative glenoid BMD on aTSA outcome overall and glenoid implant survival in particular. Finally, the methods and results presented here might eventually be implemented in preoperative planning softwares, and used to aid in better preparing the implantation of glenoid components in aTSA.

## Supplementary Information

**Additional file 1: Supplementary material.** Univariate Cox proportional hazards regression and Forest plot.

## Data Availability

The datasets used and/or analyzed during the current study are available from the corresponding author on reasonable request.

## Abbreviations

aTSA: Anatomic total shoulder arthroplasty
BMD: Bone mineral density
BMI: Body mass index
CO: Cortical bone
CT: Computed tomography
CTn: Computed tomography number
CTR: Control
HR: Hazard ratio
HU: Hounsfield unit
LSG: Loosening
OA: Osteoarthritis
SC: Subchondral cortical plate
ST: Subchondral trabecular bone
TSA: Total shoulder arthroplasty
T1-T3: Layers 1–3 of trabecular bone
VOI: Volume of interest
