# Supplementary figures and images for: Is preoperative glenoid bone mineral density associated with aseptic glenoid implant loosening in anatomic total shoulder arthroplasty?
Source: BMC Musculoskelet Disord. 2021 Jan 8;22:49. doi: 10.1186/s12891-020-03892-0 (PMC7792203; doi:10.1186/s12891-020-03892-0)

**Supplemental material**

**Univariate Cox proportional hazards regression and Forest plot**


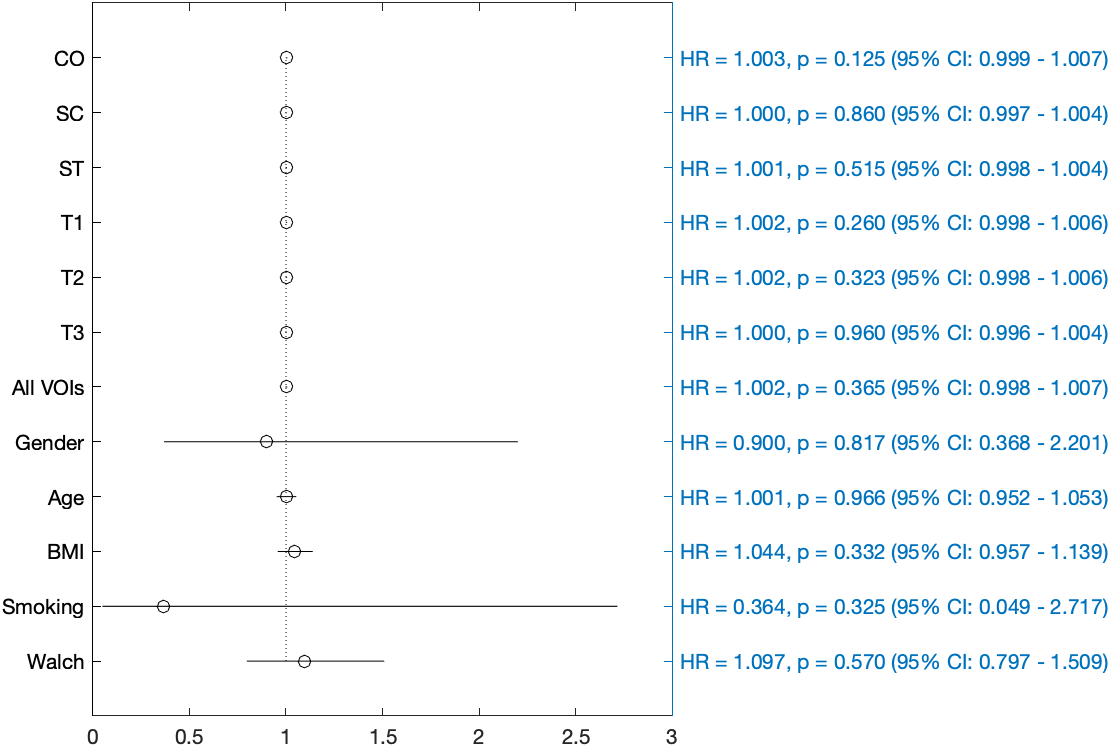

Supplement: Supplementary file 1 — Additional file 1: Supplementary material. Univariate Cox proportional hazards regression and Forest plot. [file 12891_2020_3892_MOESM1_ESM.docx]
